# Review of multiple sclerosis: Epidemiology, etiology, pathophysiology, and treatment

**DOI:** 10.1097/MD.0000000000037297

**Published:** 2024-02-23

**Authors:** Maha Haki, Haeder A. AL-Biati, Zahraa Salam Al-Tameemi, Inas Sami Ali, Hany A. Al-hussaniy

**Affiliations:** a Department of Pharmacy, Bilad Alrafidain University College, Diyala, Iraq; b Dr. Hany Akeel Institute, Iraqi Medical Research Center, Baghdad, Iraq; c Department of Pharmacology, College of Medicine, University of Baghdad, Baghdad, Iraq.

**Keywords:** cholinergic antagonists, erectile dysfunction, multiple sclerosis

## Abstract

Multiple sclerosis (MS) is a chronic autoimmune disease with demyelination, inflammation, neuronal loss, and gliosis (scarring). Our object to review MS pathophysiology causes and treatment. A Narrative Review article was conducted by searching on Google scholar, PubMed, Research Gate about relevant keywords we exclude any unique cases and case reports. The destruction of myelinated axons in the central nervous system reserves this brunt. This destruction is generated by immunogenic T cells that produce cytokines, copying a proinflammatory T helper cells1-mediated response. Autoreactive cluster of differentiation 4 + cells, particularly the T helper cells1 subtype, are activated outside the system after viral infections. T-helper cells (cluster of differentiation 4+) are the leading initiators of MS myelin destruction. The treatment plan for individuals with MS includes managing acute episodes, using disease-modifying agents to decrease MS biological function of MS, and providing symptom relief. Management of spasticity requires physiotherapy, prescription of initial drugs such as baclofen or gabapentin, secondary drug options such as tizanidine or dantrolene, and third-line treatment such as benzodiazepines. To treat urinary incontinence some options include anticholinergic medications such as oxybutynin hydrochloride, tricyclic antidepressants (such as amitriptyline), and intermittent self-catheterization. When it comes to bowel problems, one can try to implement stool softeners and consume a high roughage diet. The review takes about MS causes Pathophysiology and examines current treatment strategies, emphasizing the advancements in disease-modifying therapies and symptomatic treatments. This comprehensive analysis enhances the understanding of MS and underscores the ongoing need for research to develop more effective treatments.

## 1. Introduction

**Figure 1.**
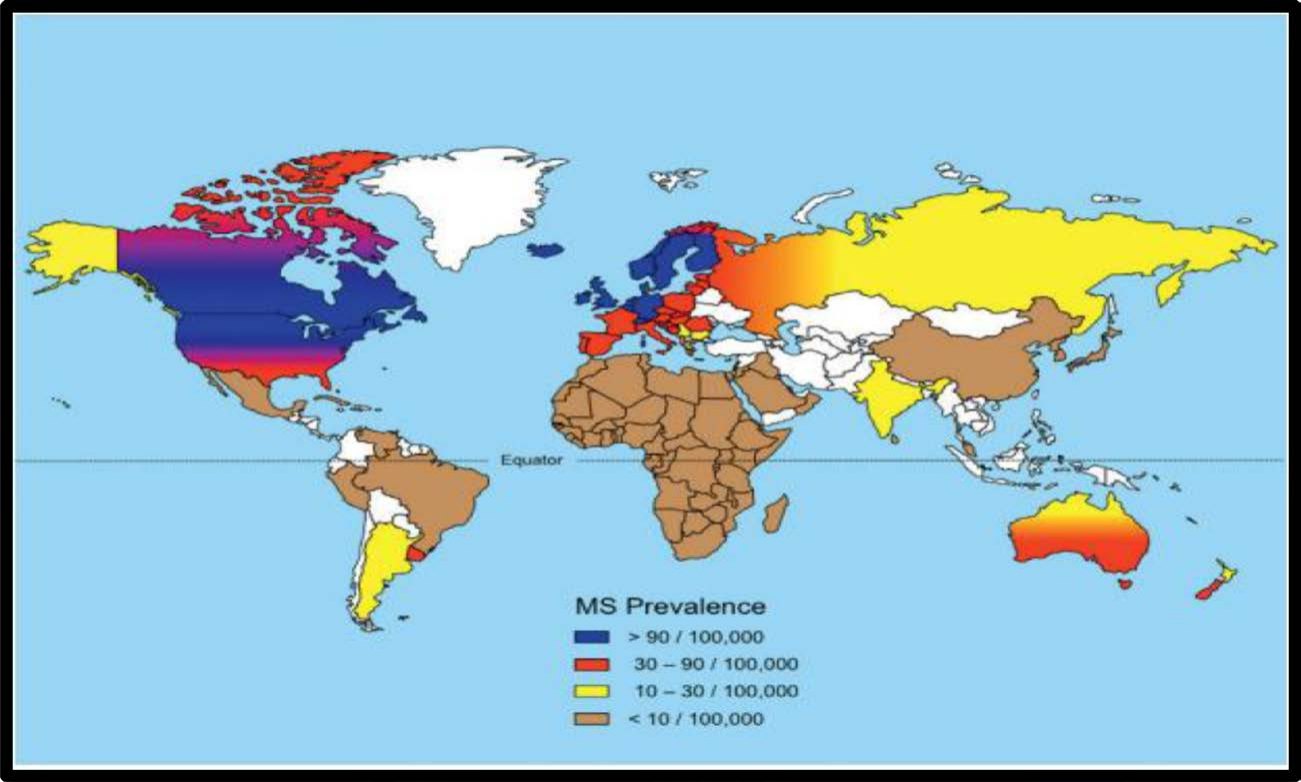
Distribution of MS prevalence. MS = multiple sclerosis.

Inflammation, demyelination, gliosis, and neuronal loss are all components of multiple sclerosis (MS), a chronic autoimmune disease that affects the central nervous system (CNS). Myelinated axons in the CNS are the target of MS attacks, which can cause varying degrees of damage to both myelin and axons.^[1,2]^ When new or recurrent neurological symptoms occur, it is referred to as clinical relapse, provided that they last for at least 24 consecutive hours without concurrent illness or fever, followed by a period of 30 days with either stability or improvement. MS affects 2.3 million people worldwide. MS is often diagnosed between the ages of 20 and 50 years, with females experiencing it more often than males.^[3,4]^ Those of Northern European descent and Whites have a higher risk of developing the disease, with a decrease in prevalence as one moves away from the equator (Fig. 1). However, certain regions have shown an increase in the occurrence of MS. The prevalence of Iraq was 4.4/100,000.^[5,6]^

## 2. Materials and methods

A Narrative Review article was conducted by searching on Google scholar, PubMed, ResearchGate about relevant keywords we exclude any unique cases and case reports.

### 2.1. Inclusion and exclusion criteria

The important criteria is only articles that published in relevant Journals, we use the Keywords and titles for looking in the internet (Google scholar and PubMed). We exclude old articles and articles that not related to our title. We also exclude articles published in predatory or not proper citable journals.

### 2.2. Ethical compliance

This is a review article not conducted on human or animal.

## 3. Result and discussion

### 3.1. Etiology of MS

MS emergence is a perplexing matter; its origins remain a mystery. However, newer investigations infer that it arises from a fusion of genetic vulnerability and environmental agents from development to young adulthood. Specific factors deemed highly culpable in regard to genetic disposition for MS and ability to make an impact include a lack of vitamin D, birth season, tobacco use, and exposure to Epstein-Barr virus^.[7,8]^

### 3.2. Pathophysiology

**Figure 2.**
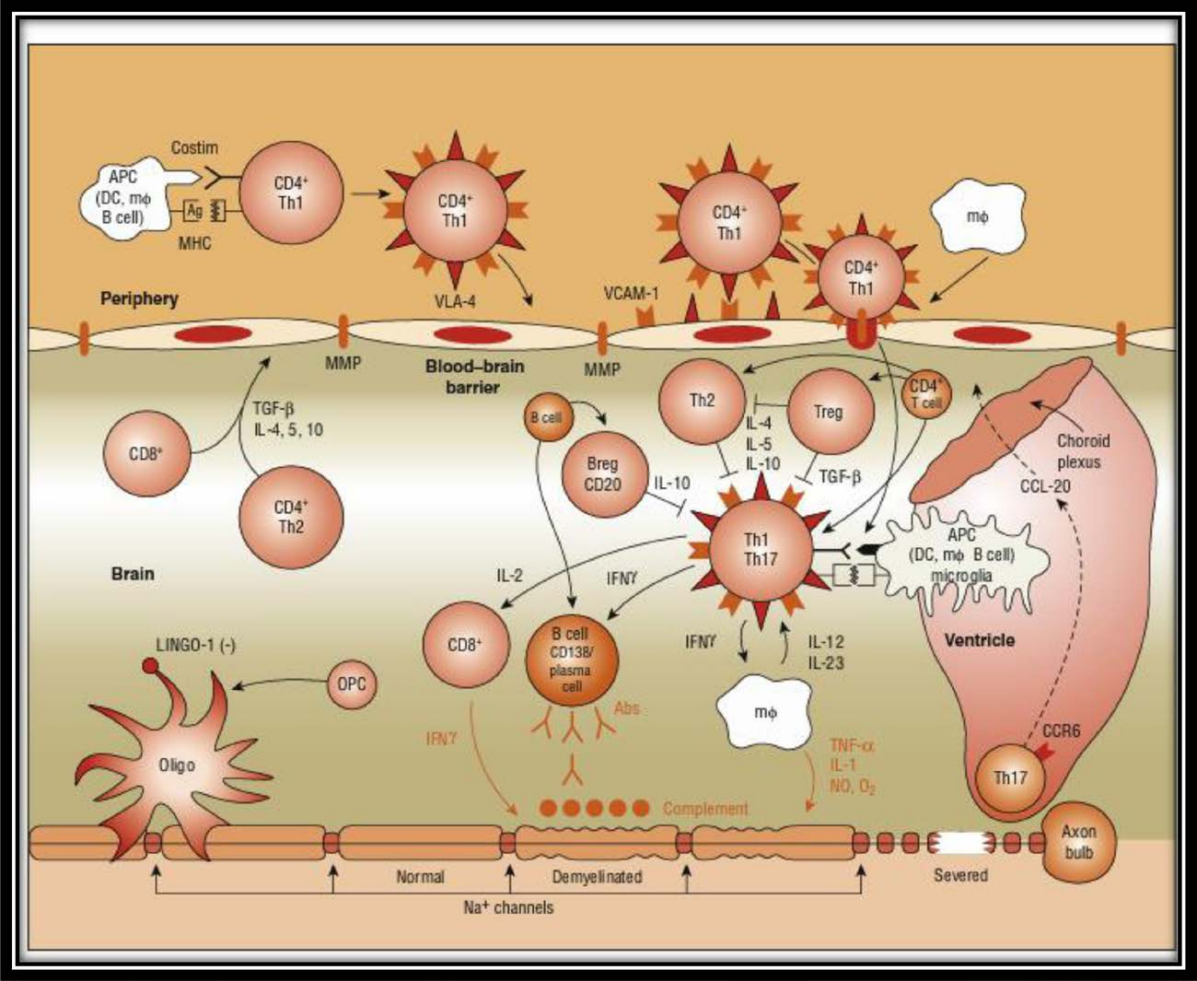
Autoimmune theory of the pathogenesis of MS.^[9]^ MS = multiple sclerosis.

T-cells can become autoreactive owing to an unknown antigen (AG) presented by major histocompatibility complex (MHC) class II molecules, leading to inflammation. This is shown in (Fig. 2).^[10,11]^

Play a crucial role in presenting AGs to T cells. Dendritic cell and macrophages (Mφ) are major antigen-presenting cells (APCs), but other cells, such as the oligodendrocyte precursor cells, also play a role. APC presentation of AGs, in the context of MHC molecules, to T cells activates these molecules. Na^+^ concentrations can affect APC function as well as matrix metalloproteinases (MMP) activity. Integrin α4β1 (very late antigen-4) on T cells binds to the Vascular Cell Adhesion Molecule-1 present on the APCs, causing T cells and APCs to migrate to AG sites. Immunoglobulin g (IgG) antibodies can also bind integrin α4β1 (very late antigen-4) and inhibit their migration.^[9]^

T cells that are autoreactive spread into lymphatic tissues and undergo expansion. They ultimately enter the circulation after being triggered by sphingosine-1-phosphate. When stimulated, T cells attach themselves to adhesion molecules that are upregulated and begin to generate MMP. MMP then causes the breakdown of the blood-brain barrier. In the CNS, they come into contact with APCs and begin dividing. Myelin is under attack, and T-helper cells develop into 2 types: proinflammatory T helper cells1 (Th1) cells and anti-inflammatory Th2 cells. Th2 cells are responsible for releasing cytokines that attack Mφ and microglial cells. Autoreactive T-cells initiate the production of B-cell antibodies that cross the damaged section of the blood-brain barrier, essentially triggering the formation of myelin autoantibodies.^[12]^

Additionally, B-cell antibodies initiate the complement cascade, which attacks myelin again. Inflammatory processes most likely cause these relapses. Targeting inflammatory processes is the focus of all the existing MS treatments.

### 3.3. 2-Degeneration

Nerve signals are disrupted by transection and axonal injury, which leads to MS progression. As early as 2 weeks after diagnosis, cytotoxic T-cells can cause axonal injury and continue to do so throughout the disease, according to growing evidence.^[12]^ This axonal loss is believed to be the primary cause of MS progression.

In MS, cytokine-producing T cells with a preference for myelin are frequently immunogenic, resulting in Th1-like proinflammatory responses. Myelin destruction in MS appears to be primarily initiated by T-helper cells (cluster of differentiation 4 [CD4]+). In particular, autoreactive CD4 + cells, particularly the Th1 variant, appear to be the main culprits and are likely activated after viral infection in the periphery. The activation of T and B cells requires 2 signals. Two signals are required for T-cell activation. The first signal involves the interaction between APC such as Mφ, B-cells, dendritic cells, and MHC. T-cell activation also requires a second signal, which is the binding between CD28 on the T-cell and B7 on APC. The interaction between T-cell CD40L and APC CD40 aids in the proliferation of B cells within the blood-brain barrier after T-cell infiltration. Peripheral T-cells attach to and roll on endothelial cells of the blood-brain barrier with the help of adhesion molecules. Post-activation, T-cells secrete MMP, aiding in the creation of openings in the blood-brain barrier to allow the entry of T-cells into the CNS. Upon entry, proinflammatory cytokines such as ILs 1, 2, 12, 17, 23, TNF-α, and interferon gamma are produced by T-cells, further opening the blood-brain barrier to allow entry of Mφ, complement, B cells, and antibodies. Activation of T cells within the CNS results in increased production of proinflammatory cytokines and potential mediators of CNS damage, such as reactive oxygen intermediates and nitric oxide. This interaction occurs with the resident microglia, astrocytes, and Mφ. Additionally, the regulation of cytokines, such as IL-4, IL-5, IL-10, and TGF-β, by CD4+, CD8+, and Th1-cells has been described. New pathogenic mechanisms, including T-cell entry via the CCR6-CCL20 axis and inhibition of myelination/demyelination via key receptor ligands (e.g., LINGO-1/NOGO66/p75 or TROY complex, Jagged-Notch signaling), have also been identified.^[13,14]^

Clinical manifestations: The weakened state of the limbs, Ataxia, Diplopia, Sensory Symptoms, Cognitive Dysfunction, Depression, Fatigue, Sexual Dysfunction, Spasticity, Optic Neuritis, Bladder Dysfunction, Constipation, Anxiety, Lhermitte symptoms, ancillary symptoms, and paroxysmal symptoms^.[15]^

**Figure 3.**
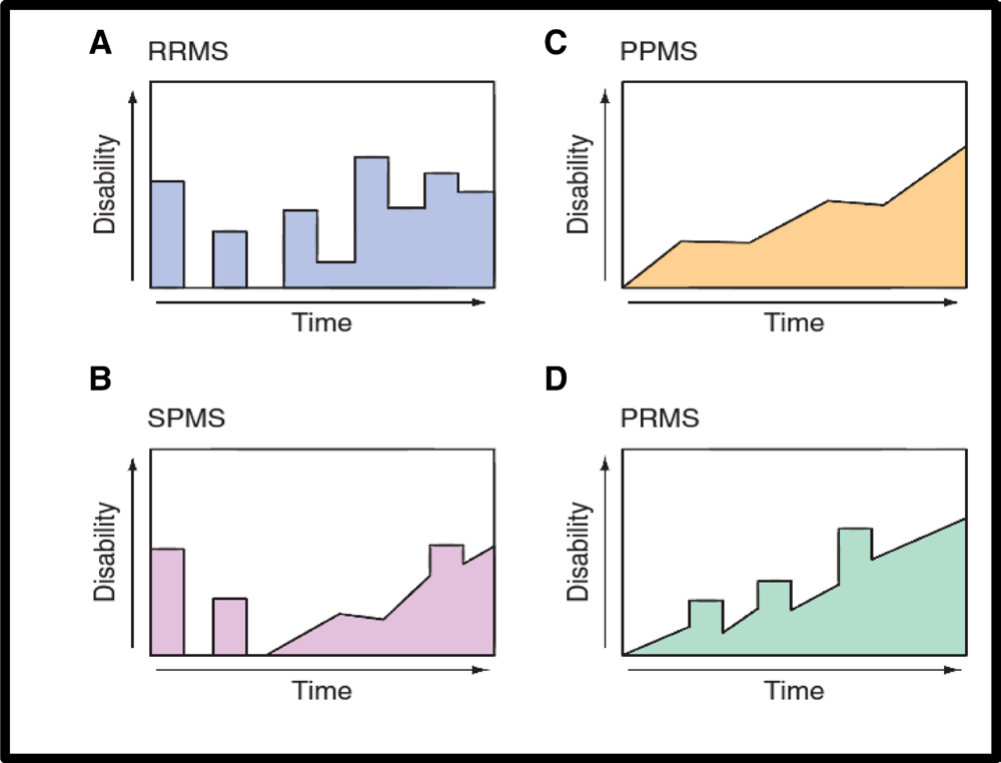
Clinical course of multiple sclerosis (MS).^[16]^ (A) Relapsing/remitting MS (RRMS), (B) secondary progressive MS (SPMS), (C) primary progressive MS (PPMS), (D) progressive/relapsing MS (PRMS).

Disease course: There are 4 major categories based on the course of the disease. Four clinical types of MS (Fig. 3).^[16]^

(1) Relapsing/remitting MS (RRMS) Discrete attacks that last days to weeks (occasionally hours) characterize RRMS, which represents 85% of MS cases at onset. Substantial or complete recovery often follows the initial attack, but this can become less evident as the attacks persist over time (see Fig. 3A). Neurological stability occurs during the period between attacks. Patients typically present with this condition.

(2) Secondary progressive MS (SPMS) As depicted in Figure 3B, the onset of SPMS is always preceded by RRMS. This transition is characterized by a constant deterioration in function that is not linked to sudden attacks, although these may or may not persist during the progressive phase^[17]^ Unlike the relatively mild and transient neurological disabilities associated with RRMS, SPMS exhibits more severe and permanent symptoms. Typically, patients with RRMS have an approximately 2% risk of transitioning to SPMS each year, indicating that most RRMS cases progress to SPMS. It seems that SPMS is just another phase of the same illness that is behind RRMS and occurs later in disease progression^.[17]^

(3) Primary progressive MS (PPMS) prevalence for PPMS disease is about 15% of the total MS causes; the disease in this type has a relatively lower frequency of attacks, but there is a decline in the study function (Fig. 3C). In comparison to the RRMS gender distribution, MS starts later in life (mean age ~40 years), and patient motor disabilities can occur faster (at least relative to the onset of the first clinical symptom). However, the changes that differentiate these 2 types of PPMS appear to represent the same underlying illness as RRMS.^[18]^

(4) MS patients with progressive/relapsing condition are rare, accounting for only about 5% of MS patients. Although they share some similarities with PPMS patients, as they both face a steady deterioration since the onset of the disease, what sets progressive/relapsing MS patients apart is the occasional attacks they experience, which is characteristic of SPMS patients (Fig. 3D). There is no shoe-fits-all when it comes to the course of MS, and each individual journey is unique.^[19]^ Recent research has revealed that the crude incidence of MS falls within the range of 31 to 85 per 100,000 individuals in many Middle Eastern countries despite traditionally being designated as low-risk zones for the disease by the Kurtzke classification. Moreover, life expectancy in the MS population, if not treated with disease-modifying therapy, may be curtailed by 8 to 12 years compared with the normal population.

Diagnosis: There is no clear diagnostic procedure. Documentation of 2 or more symptomatic episodes and 2 or more indicators of disease in physically disjointed CNS white matter tracts are diagnostic requirements for the diagnosis of definite MS^20^ (Table 1). The symptoms must be present for more than 24 hours and come in consecutive bouts separated by at least 1 month.^[21]^

**Table 1:** McDonald criteria for multiple sclerosis (MS).^[20]^

Clinical presentation	Additional data needed for MS diagnosis
2 or more assaults; 2 or more lesions determined by objective medical evidence; or 1 lesion determined by objective medical evidence along with believable historical proof of a preceding attack.	None
2 or more attacks; objective clinical evidence of 1 lesion.	Dissemination in space, demonstrated by: • ≥1 T2 lesion on MRI in at least 2 out of 4 MS-typical regions of the CNS (periventricular, juxtacortical, infratentorial, or spinal cord). OR • Await a further clinical attack implicating a different CNS site.
1 attack; objective clinical evidence of 2 or more lesions.	Simultaneous occurrence of asymptomatic gadolinium-enhancing and non-enhancing lesions at any time serves as evidence of temporal dissemination. OR • A new T2 and/or gadolinium-enhancing lesion or lesions on a subsequent MRI, regardless of when it first appeared in relation to a baseline scan. • Hold off until a subsequent clinical attack.
1 attack; objective clinical evidence of 1 lesion (clinically isolated syndrome).	The spread in space and time is shown by: • At least one T2 lesion in at least 2 of the 4 CNS areas that are characteristic for MS (periventricular, juxtacortical, infratentorial, or spinal cord) OR • Watch for a subsequent clinical episode that involves a different CNS location. AND For timely distribution: • The simultaneous existence of asymptomatic lesions that both enhance and do not enhance with gadolinium. OR • A new T2 and/or gadolinium-enhancing lesion or lesions on a subsequent MRI, regardless of when it first appeared in relation to a baseline scan. OR •delay till a subsequent clinical attack
Insidious neurologic progression suggestive of MS (PPMS).	One yr of illness onset (retrospectively or prospectively determined). PLUS 2 of the 3 aforementioned prerequisites There is evidence that the illness has spread across the brain based on one or more T2 + lesions in the MS-specific periventricular, juxtacortical, or infratentorial areas. There is proof of spatial spread in the spinal cord from 2 T2 + lesions in the cord. Positive CSF (indicators of oligoclonal bands include higher IgG index and/or isoelectric focusing).

CNS = central nervous system, CSF = cerebrospinal fluid, IgG = immunoglobulin g, MRI = magnetic resonance imaging, PPMS = primary progressive multiple sclerosis.

### 3.4. Diagnostic tests

(1) Magnetic resonance imaging (MRI): Clinical management and scientific investigation of MS have greatly benefited from MRI, which has become the primary diagnostic and monitoring aid. Conventional MRI techniques, such as spin-echo T2-weighted, pre- and post-gadolinium-enhanced spin-echo T1-weighted, and fluid-attenuated inversion-recovery images, are employed to evaluate apparent lesions and CNS atrophy. Advanced MRI techniques, including diffusion-weighted imaging, magnetization transfer imaging, magnetic resonance spectroscopy, and functional MRI, play a vital role in the pathogenesis of MS.^[20,22]^

The McDonald criteria establish that a diagnosis of MS can be made in patients who experience a CIS for the first time if demyelinating lesions with dissemination of lesions in space and dissemination of lesions in time are displayed on their brain MRIs.^[23,24]^ Brain MRIs must meet certain criteria to satisfy Barkhof-Tintore criteria and show dissemination of lesions in space.^[25,26]^ Meanwhile, dissemination of lesions in time necessitates the discovery of fresh T2 lesions in contrast to a reference MRI that was performed no less than 1 month after the start of symptoms or the presence of an asymptomatic gadolinium-enhancing lesion in an MRI administered at least 3 months after onset.^[25,26]^

(2) Evoked potentials: EP testing evaluates the performance of efferent (motor) or afferent (visual, auditory, and somatosensory) CNS circuits.^[27]^

(3) Cerebrospinal fluid: Pleocytosis of mononuclear cells and an elevated amount of intrathecal IgG are 2 CSF abnormalities associated with MS. The total protein in the CSF is typically normal. Distinguishing intrathecal synthesized IgG from passively entered serum IgG can be accomplished by using diverse formulas. The CSF IgG index is one such formula that calculates the ratio of IgG to albumin in the CSF divided by the same ratio in the serum. To determine the rate of CNS IgG synthesis, we used the IgG synthesis rate formula and measured the serum, CSF IgG, and albumin levels. Another option is to examine oligoclonal bands (OCBs) in the CSF via agarose gel electrophoresis to assess intrathecal IgG production. In over 3-quarters of patients with MS, 2 or more distinct OCBs are detected without a corresponding serum sample. Although OCBs may not appear during the onset of MS, they may increase in number over time in some individuals. Approximately a quarter of cases, primarily young patients with RRMS, exhibit mild CSF pleocytosis (>5 cells/μL). However, pleocytosis of > 75 cells/μL, the manifestation of polymorphonuclear leukocytes, or a protein concentration > 1 g/L (>100 mg/dL) in the CSF should elicit concern regarding the possibility of an MS diagnosis.^[27]^

MS treatment: There are various types of MS treatment.

1. Managing severe attacks.
2. Disease-modifying therapies that lessen MS biological activity.
3. Treatment for symptoms.

Physiotherapy, baclofen, and gabapentin are first-line medications for spasticity, tizanidine and dantrolene are second-line medications, and benzodiazepines are third-line medications.

Urinary frequency and incontinence can be treated with tricyclic antidepressants, such as amitriptyline, oxybutynin hydrochloride, and intermittent self-catheterization. Symptoms of the intestines: high-roughage diet and stool softeners. Papaverine and sildenafil were injected intracavernosally to treat impotence. Anticonvulsants are used to treat paroxysmal episodes of altered feelings. Anticonvulsants for seizures. Clonazepam was administered for cerebellar tremors.

Amitriptyline is effective for neurogenic pain as well as depression.

Amantadine 100 mg bid aids 40% of patients who are fatigued.

Currently, there are no therapies that support remyelination or brain repair, but a number of potential strategies are being intensively researched.^[28,29]^

### 3.5. Treatment of acute relapses

(1) Corticosteroids: The mechanism of action of corticosteroids may involve:

Prevention of inflammatory cytokine activation, inhibition of T and B cell activation, and prevention of immune cells from entering the CNS.^[30]^ Corticosteroids accelerate functional recovery.^[31]^ Equal efficacy of equivalent doses of IV and oral dosage forms has been shown with oral dosing, avoiding discomfort, inconvenience, and the expense of IV therapy.^[32]^ Adverse Effects: The common adverse effects were gastrointestinal upset, insomnia, hypertension, osteoporosis, hyperglycemia, and mood disturbances.^[32]^

Dosing and administration: Methylprednisolone dose is 500–1000 mg/d IV as one dose or in divided doses. Oral prednisone (1250 mg/d) or oral methylprednisolone (1000–1250 mg/d) provided an equivalent dose. These regimens were given for 3 to 7 days. Clinical improvement usually begins with therapy. Recovery was equal with or without subsequent oral corticosteroid tapering.^[33]^

**Figure 4.**
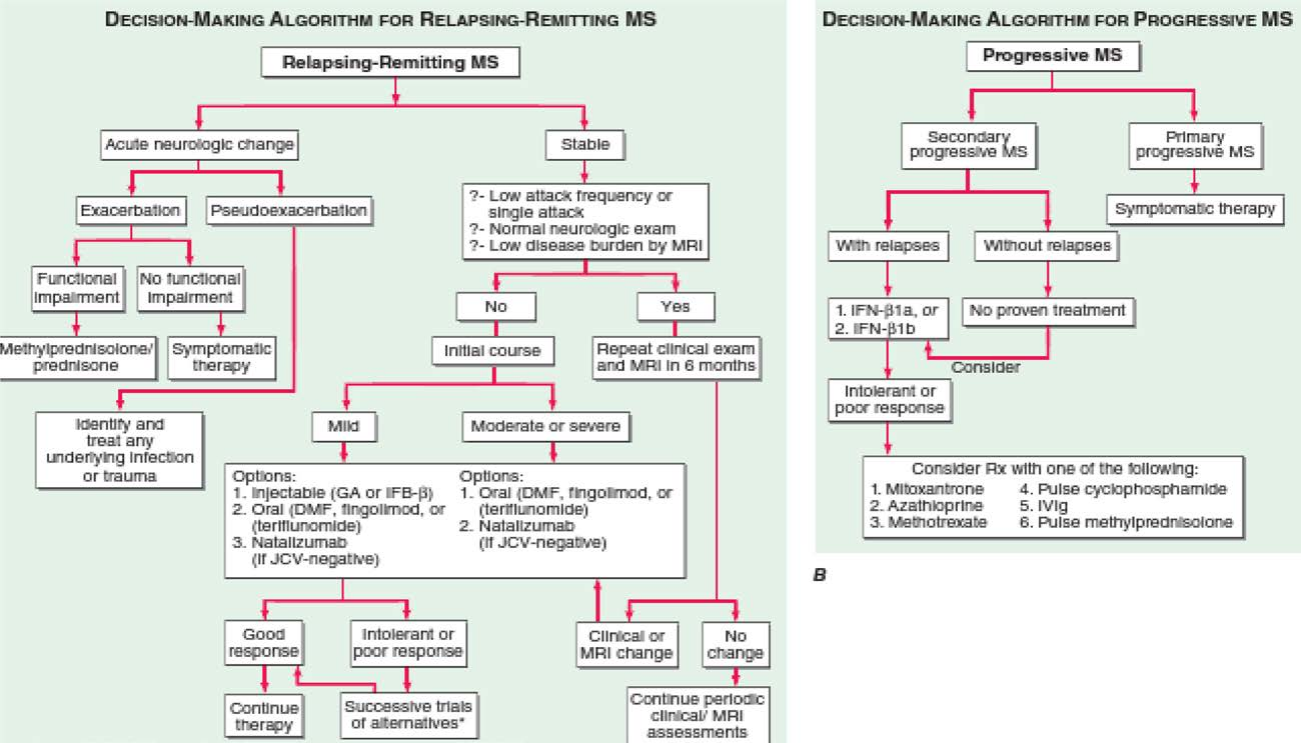
Multiple sclerosis treatment selection.^[36]^ It is possible to conduct studies of various interferon (IFN-) preparations, particularly moving from an Avonex-style once-weekly dose schedule to one that involves more frequent dosing (e.g., Rebif, Betaseron/Extavia). Additionally, natalizumab usage is an option for people who have the JC virus. The MRI, or magnetic resonance imaging. MRI = magnetic resonance imaging.

(2) Plasma exchange: Patients who experience fulminant attacks that do not respond to glucocorticoids may benefit from 5 to 7 exchanges:40 to 60 mL/kg each exchange given every other day for 14 days. However, the price is considerable, and there is not enough solid proof of its effectiveness.^[34–38]^

(3) Disease-modifying therapies: Injectable medications (IFN- or glatiramer acetate) have been traditionally used as the first-line treatment for the majority of patients with relapsing types of MS. This has started to change, however, with the development of potent and likely secure oral medications such as dimethyl fumarate, fingolimod, and teriflunomide. Additionally, natalizumab, a monthly infusion medication that is extremely successful, well tolerated, and seemingly safe in patients who do not have JC antibodies, offers an appealing alternative in many circumstances.^[39,40]^ Clinicians must base their decisions on the best available evidence as well as pragmatic factors^.[41–46]^ One sensible strategy is to divide illness aggressiveness into 2 stages before making the initial decisions (Fig. 4).

Currently, 13 medications are approved by the European Medicine Agency and Food and Drug Administration as disease-modifying treatments for MS, including IFN-1a (Avonex), IFN-1b (Betaseron or Extavia), glatiramer acetate (Copaxone), natalizumab (Tysabri), fingolimod (Gilenya), and dimethyl fumarate (Tecfi).^[9,46–49]^ Numerous studies have examined the effect of clinical factors (relapses and the development of impairment) and/or MRI activity (defined as either new or expanding T2-lesions relative to baseline MRI scans or new or increased gadolinium-enhancing lesions) to identify disease-modifying treatment responders and non-responders.^[50–54]^ IFN-Bs, glatiramer acetate therapy, teriflunomide, and dimethyl fumarate, are currently recommended by the Middle East and North Africa Committee for Treatment and Research in Multiple Sclerosis guidelines as first-line therapies for RRMS, and fingolimod is a suitable substitute for patients with needle phobia or other contraindications to the aforementioned drugs, for patients with a severely active illness (two or more debilitating relapses in a year associated with MRI abnormalities), fingolimod, natalizumab, or alemtuzumab are recommended as second-line treatments.^[55–58]^

Evidence from randomized control trials and real-world observational studies suggests that switching from first-line therapies to second-line treatments (natalizumab, fingolimod, alemtuzumab, and ocrelizumab) after first-line therapies have failed to control inflammatory activity is typically associated with a better level of control.^[57,58]^

## 4. Conclusion

The review takes about MS causes Pathophysiology and examines current treatment strategies, emphasizing the advancements in disease-modifying therapies and symptomatic treatments. This comprehensive analysis enhances the understanding of MS and underscores the ongoing need for research to develop more effective treatments.

## Acknowledgments

We thank the Iraqi Medical research center (Dr Hany Akeel Institute) for their great support to our work.

## Author contributions

**Conceptualization:** Zahraa Salam Al-Tameemi, Hany A. Al-hussaniy.

**Data curation:** Hany A. Al-hussaniy.

**Formal analysis:** Inas Sami Ali.

**Resources:** Maha Haki.

**Software:** Zahraa Salam Al-Tameemi.

**Validation:** Haeder A. AL-Biati, Zahraa Salam Al-Tameemi, Inas Sami Ali.

**Visualization:** Haeder A. AL-Biati, Zahraa Salam Al-Tameemi, Inas Sami Ali, Hany A. Al-hussaniy.

**Writing – original draft:** Maha Haki, Haeder A. AL-Biati, Hany A. Al-hussaniy.

**Writing – review & editing:** Inas Sami Ali, Hany A. Al-hussaniy.
